# Asset pricing implications of good governance

**DOI:** 10.1371/journal.pone.0214930

**Published:** 2019-04-05

**Authors:** Thorsten Lehnert

**Affiliations:** Luxembourg School of Finance, University of Luxembourg, Luxembourg, Luxembourg; The Bucharest University of Economic Studies, ROMANIA

## Abstract

In this paper, I aim to explore the effect of good governance on equity returns, and empirically investigate if governance at the country level has asset pricing implications and contributes to the idiosyncrasy of price jumps. Jumps are found to be far less systematic than the smooth (non-jump) component of country price indexes. Hence, if jumps are more idiosyncratic, governance should primarily affect the jump risk component. This is good news for international investors, because diversification provides insurance against jumps. Relying on an equilibrium asset-pricing model in an economy under jump diffusion, I decompose the moments of the returns of international stock markets into a diffusive (systematic) risk and a (idiosyncratic) jump risk part. For a balanced panel of 52 countries, my results suggest that governance is an important determinant of (idiosyncratic) jump risk. Stock markets in poorly governed countries are characterized by higher volatility and more negative return asymmetry, primarily driven by the higher jump risk. Regulatory quality, the government effectiveness and the control of corruption appear to be most important. Results are robust to the inclusion of various controls for other country- or market-specific characteristics.

## Introduction

The work of La Porta et al. [1–3] suggest that the legal environment, as described by both legal rules and their enforcement, matters for the size and extent of a country's capital markets. Because a good legal environment protects the potential financiers against expropriation by entrepreneurs, it raises their willingness to surrender funds in exchange for securities, and hence expands the scope of capital markets and contributes positively to the development of financial markets. Johnson and Shleifer [4] illustrate the centrality of law enforcement in making financial markets work, and the possible role of regulators in law enforcement. Lombardo and Pagano [5] investigate whether international differences in the quality of government institutions also help explain the international cross-section of expected stock returns. They find that total stock market returns are positively correlated with overall measures of the quality of institutions, such as judicial efficiency and rule of law. They interpret the positive effect of the overall quality of institutions on equity returns as capturing the resulting curtailment of private benefits and increase of profitability, under imperfect international integration of stock markets. Aggarwal et al. [6] find that fund managers invest less in countries with poor legal environments and low governance standards. Furthermore, Lombardo and Pagano [7] argue that the existence of this superior risk-return relationship for countries with better institutions is supported under international market segmentation providing diversification benefits. Rodríguez-Pose and Garcilazo [8] examine the impact of the quality of local and regional governments on the returns of investment. Their findings suggest that the quality of government is an important determinant of economic growth, but also a key factor determining the returns of public investment. Regions that receive structural funds only experience an improvement in economic growth when the quality of government is significantly enhanced. The findings of Hooper et al. [9] suggest that countries with better-developed governance systems have stock markets with higher returns on equity and lower levels of risk. Hail and Leuz [10] investigate international differences in firms cost of equity capital and analyze whether the effectiveness of a country’s legal institutions and securities regulation is systematically related to cross-country differences in the cost of equity capital. They find that countries with extensive securities regulation and strong enforcement mechanisms exhibit lower levels of cost of capital than countries with weak legal institutions.

Despite the importance of governance affecting the content of information transmission in markets and society, previous literature provides no guidance on how cross-country differences in governance affect the news-returns relationship in stock markets. According to the growth-enhancing governance approach, good governance fosters economic growth by managing incentives to enhance productivity and help shift activity to more economically productive activities. The market-enhancing governance approach assesses the effects of good governance by linking governance and economic outcomes. Effective governance fosters strong property and contract rights and a stable rule of law, which keeps transaction costs low to permit private individuals and entities to increase their own utility and economic potential. Risk Governance is a closely related concept. According to the International Risk Governance Council, “Governance refers to the actions, processes, traditions and institutions by which authority is exercised and decisions are taken and implemented. Risk governance applies the principles of good governance to the identification, assessment, management and communication of risks. Better risk governance implies enabling societies to benefit from change, while minimizing the negative consequences of the associated risks.” For any country-level statistical analysis on governance, researchers typically make use the six composite measures proposed and reported as part of the World Bank Worldwide Governance Indicators (WGI) Project21. Four measures are primarily concerned with the quality of the delivery of government services: government effectiveness, regulatory quality, rule of law, and the control of corruption. The other two indicators measure the state of democracy: the first covering voice and accountability, and the second political stability and absence of violence. Using all six World Bank worldwide governance indicators, Helliwell et al. [11] investigate the empirical linkages between good governance and national well-being. They find that people are more satisfied with their lives in countries having better governance quality. Furthermore, actual changes in governance quality since 2005 have led to large changes in the quality of life. They interpret this as strong evidence that governance quality can be changed, and that these changes have much larger effects than those flowing through a more productive economy. Regarding the quality of governance services, researchers empirically investigate the impact of corruption on investment and economic growth. Haque and Kneller [12] argue that corruption reduces the quality of public services, but inflates the public spending. However, it reduces the returns to public investment and makes it ineffective in raising economic growth. Cieślik and Łukasz [13] find that corruption directly hinders economic growth by hampering investment. Evidently, bribes, unlike taxes, involve unpredictable distortion in the discretionary and uncertain use of the government power. The uncertainty induced by corruption acts similarly as a tax on entrepreneurship and productive action. Importantly, they show that corruption decreases the return on investment and increases its variance, which discourages investment activities. Chiou et al. [14] examine how the legal environment in a country influences performance and risk of stock across countries at different developmental stages. They confirm that equities in countries with English common law origin have higher risk premiums than those in civil law countries, particularly for countries of the French/Spanish code. The indicators representing high efficiency in law system, low corruption, strong legal protection of investors' rights, and reliable political environment are associated with low risk and high performance.

In recent year, a number of empirical and theoretical studies find evidence for the existence of jumps and their substantial impact (see e.g. Johannes [15]). Risk compensation principles suggest that price jumps can be considered to be a risk and risk-averse investors that invest in the stock market want to be compensated for accepting this risk. Eraker et al. [16] find that jumps command larger risk premiums than continuous returns. Researchers rely on jump detection methods to investigate the news-returns relationship in financial markets. They find that positive (negative) jumps are a result of good (bad) economic news. Not surprisingly, results further suggest that the response is asymmetric: bad news has a larger impact on returns than good news. Rangel [17] finds that, when differentiating between ‘‘normal” and ‘‘surprising” news events, jumps are more frequent on announcement days compared to non-announcement days. For equity, bond and currency markets, the findings of Evans [18] suggest that about one-third of the observed price jumps occur on days of macroeconomic news announcements. Again the “informational surprise” component contained in the announcements affects the jump sizes positively. Furthermore, Jiang et al. [19] investigate the importance of macroeconomic news announcements compared to liquidity shocks. Using 5-minute price data on Treasury notes and bonds, they conclude that announcement shocks play an important role in the price discovery process. Despite the importance of price jumps, previous literature provides little guidance about the international nature of jumps in stock markets and its relationship with country characteristics. The extent of international correlation is very important for diversifying investors and government officials attempting to coordinate policies across borders. Government fiscal and monetary authorities should be interested in jump correlations, which have implications for international policy coordination. Returns on international equities are typically characterized by jumps, which might be more prominent in emerging market returns (Bekaert et al. [20]), but tend to occur at the same time across countries leading to systemic risk (Das and Uppal [21]). Asgharian and Bengtsson [22] study jump spillover in international equity markets. Their results suggest that significant jumps in large markets lead to jumps in other markets. Markets in the same region and with similar industry structures typically experience jump contagion. Using various measures of jumps and data for a set of countries over several decades, Pukthuanthong and Roll [23] present empirical evidence that price jumps are prevalent in stock markets of most countries. Their general finding is that jumps are correlated across countries but they are less correlated than returns. Jumps are less systematic than the smooth (non-jump) component of country price indexes. Lee [24] finds that U.S. jumps are mostly attributable to events such as Federal Reserve announcements or initial jobless claims, which are mainly idiosyncratic from a global perspective.

In this paper, I aim to explore the effect of good governance on equity returns, and empirically investigate if governance at the country level has asset pricing implications and contributes to the idiosyncrasy of price jumps. Jumps are found to be far less systematic than the smooth (non-jump) component of country price indexes. Hence, if jumps are more idiosyncratic, governance should primarily affect the jump risk component. This is good news for international investors, because diversification provides insurance against jumps. Relying on an equilibrium asset-pricing model in an economy under jump diffusion, I decompose the moments of the returns of international stock markets into a diffusive (systematic) risk and a (idiosyncratic) jump risk part. Using stock market data for a balanced panel of 52 countries, my results suggest that the regulatory quality, the government effectiveness and the control of corruption are important determinant of (idiosyncratic) jump risk. Stock markets in poorly governed countries are characterized by higher volatility and more negative return asymmetry, primarily driven by the higher jump risk. Results are robust to the inclusion of various controls for other country- or market-specific characteristics. Therefore, our results lend further support for the view that a precondition for financial market development is the improvement of the government institutions.

## Materials and methods

In order to decompose stock price risk into a diffusive part and a jump risk part, I rely on the jump-diffusion model in a production economy of Zhang et al. [25]. The price of an asset *S*_*t*_ (the market portfolio) can be assumed to follow the following process:

$$\frac{{dS}_{t}}{S_{t}}=(r_{f}+\phi )dt+\sigma dB_{t}+(e^{x}-1)(dN_{t}-\lambda dt)$$

where *r*_*f*_ is the risk-free rate, *ϕ* represents the excess market return, *σ* denotes volatility of stock prices, *B*_*t*_ is a standard Brownian motion in R (and *dB*_*t*_ the increment), *N*_*t*_ is a Poisson process with constant intensity *λ* (and *dN*_*t*_ the increment) and jump size *x*. The arrival of jumps follows a Poisson process, which has *E*(*dN*_*t*_) = *λdt* with arrival intensity *λ*≥0. (*e*^*x*^−1) is the relative jump size of the rare event.

The representative investor has a constant relative risk aversion utility function as

$$U(c)=\left\{ \begin{array}{l} \frac{c^{1-\gamma }}{1-\gamma },\;\gamma >0,\gamma \neq 1\\ lnc,\;\gamma =1 \end{array}\right.$$

with *U*′(*c*)>0 and *U*′′(*c*)<0. $\gamma =\frac{-cU^{\prime \prime }(c)}{U^{\prime }(c)}$ measures the magnitude of relative risk aversion and c is consumption. The investor is assumed to allocate a fraction *w* of total wealth *W*_*t*_ in stock *S*_*t*_ and a fraction (1−*w*) in the risk-free asset and maximizes his expected utility of consumption throughout his lifetime by choosing the fraction *w* of wealth to investment and the consumption rate *c*_*t*_ at each time t. Mathematically,

$${max}_{\left(c_{t},w\right)}E_{t}\int_{t}^{T}\beta (t)U(c_{t})dt$$

subject to his wealth constraint as

$$\frac{dW_{t}}{W_{t}}=\left[r_{f}+w\phi -w\lambda (e^{x}-1)-\frac{c_{t}}{W_{t}}\right]dt+w\sigma dB_{t}+w(e^{x}-1)dN_{t}$$

where *β*(*t*)≥0 (0≤*t*≤*T*) is a time preference function and *ϕ* represents the risk premium due to investment in risky stocks. Zhang et al. [25] shows that the market excess return *ϕ* can be expressed as the sum of a diffusive risk premium, *ϕ*_*σ*_, and a jump risk premium, *ϕ*_*J*_:

$$\phi_{\sigma }=\gamma \sigma^{2}$$

$$\phi_{J}=\lambda E[\left(1-e^{-\gamma x})(e^{x}-1\right)]$$

$$\phi \equiv \mu -r_{f}\equiv \phi_{\sigma }+\phi_{J}$$

where $Y_{\tau }=\ln\left(\frac{S_{t+\tau }}{S_{t}}\right)$. I hypothesize that in countries with poor governance system, the higher frequency of negative jumps leads to a higher jump risk premium; however, there could also be an indirect on diffusive risk. In order to quantify the diffusive risk and the jump risk, the jump size *x* is assumed to be non-random. The density of daily stock market returns *r*_*τ*_ (τ=1252) is given by

$$p(r_{\tau })=\frac{1}{2\pi }\int_{-\infty }^{+\infty }Re[e^{-ikr_{\tau }}f_{r_{\tau }}(k)]dk$$

where

$$ln\left(f_{r_{\tau }}(k)\right)=ik\mu \tau -\frac{1}{2}ik(1-ik)\sigma^{2}\tau +\lambda \tau \left(e^{ikx}-1-ik(e^{x}-1)\right)$$

Countries’ stock market returns (*μ*,*σ*,*λ*,*x*) are expressed in annual terms and $f_{r_{\tau }}(k)$ is its characteristic function. Using Romberg integration methods, I evaluate the integral numerically. By maximizing the log likelihood function $L_{r_{\tau }}(\mu ,\sigma ,\lambda ,x)=\sum_{n=1}^{N}\ln\left(p_{n}(r_{\tau })\right)$, the parameters of the model can be estimated. Finally, one can derive the second and third central moments as

$${E(r_{\tau }-E(r_{\tau }))}^{2}=\tau (\sigma^{2}+\lambda x^{2})$$

$${E(r_{\tau }-E(r_{\tau }))}^{3}=\tau \lambda x^{3}$$

## Results and discussion

In my empirical analysis, I aim to investigate the relationship between governance and diffusive/jump risk in international stock markets. Based on daily data, the model parameters (*μ*,*σ*,*λ*,*x*) are estimated for each country and for each year, separately. Hence, I construct a measure of total return volatility (*TOTALRISK*) for country i’s stock market in year t, that is a combination of diffusive risk and jump risk

$${TOTALRISK}_{i,t}=\sigma_{i,t}^{2}+\lambda_{i,t}x_{i,t}^{2}$$

I hypothesize that poor governance can be associated with higher jump risk and/or higher diffusive risk in stock prices. As a result, volatility would be higher in those countries.

Additionally, I decompose total risk into a measure for diffusive risk and jump risk

$${DIFFURISK}_{i,t}=\sigma_{i,t}^{2}$$

$${JUMPRISK}_{i,t}=\lambda_{i,t}x_{i,t}^{2}$$

and separately analyze both measures. I hypothesize that in poorly governed countries, the higher frequency of negative jumps leads to higher jump risk. While the impact on jump risk can be assumed to be more direct, there could be an indirect effect on diffusive risk as well.

Furthermore, I derive a measure of return asymmetry (non-normalized skewness) that is a result of the jump risk component only

$${SKEWNESS}_{i,t}=\lambda_{i,t}x_{i,t}^{3}$$

I hypothesize that in countries with a poor governance system, the more frequent negative jumps affect the return asymmetry. As a result, stock markets in those countries are characterized by a more negative return asymmetry.

**Fig 1:**
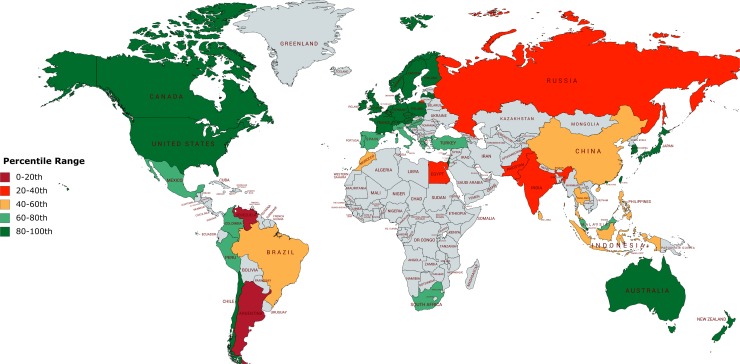
Worldwide governance indicators–regulatory quality, 2013. The figure shows the worldwide governance indicators, the regulatory quality in particular, for all countries in the sample at the end of the sample period. The figure was created by the author using *mapchart*.*net*. The colors refer to the percentile range of all countries. For example, the red color refers to countries with a poor governance system, while the green color refers to well governed countries. The 52 countries used in my study are Argentina, Australia, Austria, Belgium, Brazil, Canada, Chile, China, Colombia, Croatia, Czech Republic, Denmark, Egypt, Finland, France, Germany, Greece, Hong Kong, Hungary, India, Indonesia, Ireland, Israel, Italy, Japan, Latvia, Luxembourg, Malaysia, Mexico, Morocco, Netherlands, New Zealand, Norway, Pakistan, Peru, Philippines, Poland, Portugal, Russia, Singapore, South Africa, South Korea, Spain, Sri Lanka, Sweden, Switzerland, Taiwan, Thailand, Turkey, United Kingdom, United States, Venezuela. (Source: http://info.worldbank.org/governance).

I choose 52 international stock market indices in this study to cover a frequently used set of countries with well-functioning stock markets and a recent time period, 2002–2013. For those countries, Bartram et al. [26] show that it is possible to obtain reliable data at the daily frequency. The authors make use of a sample of 50 countries; in addition I also include Croatia and Latvia. All stock market data are from Thomson Reuters DataStream. My key explanatory variable is a series of country specific governance indicators from the World Bank Group. The World Bank governance indicators report on six broad dimensions of governance. From 2002 the indicators are available on an annual basis. According to the World Bank (For more information, see http://info.worldbank.org/governance.), “Governance consists of the traditions and institutions by which authority in a country is exercised. This includes the process by which governments are selected, monitored and replaced; the capacity of the government to effectively formulate and implement sound policies; and the respect of citizens and the state for the institutions that govern economic and social interactions among them.” These data are gathered from a number of survey institutes, think tanks, non-governmental organizations, international organizations, and private sector firms. The six composite measures are useful as a tool for cross-country comparison. All World Bank country scores are aggregate indicators, in units of a standard normal distribution, i.e. ranging from approximately -3 to 3. Higher scores relate to better governance and, therefore, characterize more developed countries. The Voice and Accountability measure (*VOICE*) captures perceptions of the extent to which a country's citizens are able to participate in selecting their government, as well as freedom of expression and freedom of association. The Control of Corruption (*CORRUP*) measure captures perceptions of the extent to which public power is exercised for private gain, including both petty and grand forms of corruption, as well as "capture" of the state by elites and private interests. The Government Effectiveness score (*GOVEFF*) captures perceptions of the quality of public services, the quality of the civil service and the degree of its independence from political pressures, the quality of policy formulation and implementation, and the credibility of the government's commitment to such policies. Political Stability and Absence of Violence/Terrorism (*POLSTAB*) captures perceptions of the likelihood that the government will be destabilized or overthrown by unconstitutional or violent means, including politically-motivated violence and terrorism. The measure Regulatory Quality (*REGQUAL*) captures perceptions of the ability of the government to formulate and implement sound policies and regulations that permit and promote private sector development. Rule of Law (*RULELAW*) captures perceptions of the extent to which agents have confidence in and abide by the rules of society, and in particular the quality of contract enforcement, property rights, the police, and the courts, as well as the likelihood of crime and violence (Description of the governance variables according to the World Bank Group. More information on www.worldbank.org). In Fig 1, I show a map highlighting all countries used in my study and their respective governance quality (here regulatory quality). For example, the red color refers to countries with a poor governance system (e.g. Argentina) and the green color refers to well governed countries (e.g. Canada).

The control variables that I use in my empirical analysis are obtained for country *i* and sample year *t*. The set of controls is in line with a comparable study by Bae et al. [27]. I make use of country- and market-specific variables. In order to account for income differences between countries, I use the logarithm of the GDP per capita in US dollar, *lnGDPPC*. GDP per Capita is typically used in similar studies. Alternatively, one could use the GDP growth rate, which is known to have a linear negative relationship with GDP per Capita. I find that qualitatively results don’t change once I use the GDP growth rate in the analysis. The logarithm of the average daily stock market turnover, *lnTURNOVER*, is used as a proxy for the size of the national stock markets. In order to control for the performance of the national stock market, the annualized average daily return, *RETURN*, of country i within a particular year t is used. Additionally, *FXVOL* controls for the possible impact of currency fluctuations on local stock markets, which denotes the annualized standard deviation of daily currency returns vis a vis the dollar of country i in year t. For the US, the euro is used instead. The United Nations Human Development Index (*HDI*), interpreted as the average achievement of a country in key dimensions of human development, is used as measure of development of country i in year t. The HDI was created to emphasize that people and their capabilities should be the ultimate criteria for assessing the development of a country, not necessarily economic growth alone. All country- and market-specific data are from Thomson Reuters DataStream. HDI data are from the United Nations Development Programme. Table 1 provides summary statistics of my dependent and independent variables over all countries and years.

**Table 1:** Descriptive statistics.

	Mean	Median	Stdev	Min	Max
*Dependent Variables*:					
*TOTALRISK*	0.050	0.035	0.048	0.004	0.392
*DIFFURISK*	0.039	0.027	0.036	0.003	0.291
*JUMPRISK*	0.012	0.007	0.015	0.001	0.154
SKEWNESS (*100)	-0.026	-0.011	0.283	-5.769	2.234
*Independent Variables*:					
*VOICE*	0.567	0.899	0.948	-2.284	1.826
*RULELAW*	0.693	0.891	1.015	-1.686	2.000
*REGQUAL*	0.764	1.059	0.957	-2.505	1.996
*POLSTAB*	0.157	0.469	1.007	-2.812	1.665
*GOVEFF*	0.832	1.012	0.980	-2.258	2.423
*CORRUP*	0.717	0.601	1.142	-1.831	2.553
*lnGDPPC*	4.132	4.278	0.553	2.684	5.049
*lnTURNOVER*	16.75	16.95	2.414	8.309	24.42
*RETURN*	0.078	0.130	0.324	-1.104	1.581
*FXVOL*	0.097	0.088	0.078	0.002	0.989
*HDI*	0.794	0.835	0.109	0.454	0.944

The Table presents summary statistics (mean, median, standard deviation, minimum and maximum) for my dependent and independent variables for all countries and years. TOTALRISK denotes the total stock market risk in country i and year t, while DIFFURISK denotes the diffusive part and JUMPRISK denotes the jump risk part. SKEWNESS is my measure of return asymmetry. The Voice and Accountability measure (VOICE) captures perceptions of the extent to which a country's citizens are able to participate in selecting their government. The Corruption (CORRUP) measure captures perceptions of the extent to which public power is exercised for private gain. The Government Effectiveness score (GOVEFF) captures perceptions of the quality of public services. Political Stability and Absence of Violence/Terrorism (POLSTAB) captures perceptions of the likelihood that the government will be destabilized or overthrown by unconstitutional or violent means. Regulatory Quality (REGQUAL) captures perceptions of the ability of the government to formulate and implement sound policies and regulations. Rule of Law (RULELAW) captures perceptions of the extent to which agents have confidence in and abide by the rules of society. lnGDPPC denotes the logarithm of the GDP per capita in US dollar. lnTURN is the logarithm of the average daily stock market turnover. RETURN refers to the annualized average daily return of country X within a particular year. FXVOL denotes the annualized standard deviation of daily currency returns vis a vis the dollar. HDI is the United Nations Human Development Index.

The average annualized stock market volatility ($\sqrt{TOTALRISK}$) across countries is around 20%, with a minimum of 7% and a maximum of 63% in a particular country and year. Not surprisingly, the diffusive contributes more to the overall stock market risk compared to jump risk. My measure of normalized skewness (*SKEWNESS*) that is a result of the jump risk component is typically negative, suggesting the existence of predominately negative jumps in stock prices. When I compare my *SKEWNESS* measures for poorly and well governed countries, I find *SKEWNESS* to be more negative in countries with low governance scores, where the difference is typically statistically significant at the 1% level.

I am testing my hypotheses using a dynamic panel data model with either *TOTALRISK*_*i*,*t*_, *DIFFURISK*_*i*,*t*_, *JUMPRISK*_*i*,*t*_, or *SKEWNESS*_*i*,*t*_ as dependent variable:

$$Y_{i,t}=\alpha_{0}+\alpha_{1}Y_{i,t-1}+\alpha_{2}\;{GI}_{i,t}+\alpha_{3}\;{lnGDPPC}_{i,t}+\alpha_{4}\;{lnTURNOVER}_{i,t}$$

$$+\alpha_{5}\;{RETURN}_{i,t}+\alpha_{6}{FXVOL}_{i,t}+\alpha_{7}{HDI}_{i,t}+\upsilon_{t}+\eta_{t}+e_{i,t}$$

Further to previously defined variables, *GI*_*i*,*t*_ refers to a particular governance indicator of country i in year t as defined earlier. *υ*_*t*_ is a time specific effect and *η*_*t*_ is an unobserved country-specific fixed effect. I account for endogeneity and country-specific unobserved characteristics by using the two-step system GMM estimator (see Blundell and Bond [28]). Standard errors are computed using the finite-sample correction suggested by Windmeijer [29]. The Sargan test of overidentification and the Arellano-Bond [30] test for autocorrelation of the residuals is used to assess the consistency of the system GMM estimator. Table 2 provides the results for a regression using the annualized aggregate stock market volatility (*TOTALRISK*) as the dependent variable.

**Table 2:** Two-step system GMM estimator, *TOTALRISK*.

*Independent* *Variables*		*VOICE* *(1)*	*RULELAW* *(2)*	*REGQUAL* *(3)*	*POLSTAB* *(4)*	*GOVEFF* *(5)*	*CORRUP* *(6)*
*GOVERNANCE INDICATOR*		-0.009 (-1.59)	-0.008 (-1.26)	-0.010** (-2.37)	-0.002 (-1.36)	-0.010*** (-2.79)	-0.009** (-2.21)
*TOTALRISK*_*t-1*_	0.317*** (6.87)	0.328*** (6.85)	0.333*** (7.58)	0.329*** (7.33)	0.311*** (6.55)	0.311*** (6.97)	0.319*** (6.78)
*lnGDPPC*	-0.030** (-2.38)	-0.029* (-2.01)	-0.026* (-1.79)	-0.029** (-2.28)	-0.025* (-1.92)	-0.029** (-2.31)	-0.029** (-2.22)
*lnTURN*	0.003** (2.23)	0.003* (1.75)	0.002* (1.69)	0.003** (2.20)	0.002* (1.81)	0.003* (1.98)	0.003** (2.16)
*RETURN*	-0.003* (-1.76)	-0.005** (-1.97)	-0.005** (-2.03)	-0.006** (-2.06)	-0.005* (-1.77)	-0.006** (-1.97)	-0.005* (-1.76)
*FXVOL*	0.034 (0.89)	0.017 (0.49)	0.012 (0.32)	0.009 (0.23)	0.038 (0.98)	0.002 (0.05)	0.005 (0.14)
*HDI*	-0.059 (-0.54)	-0.037 (-0.32)	-0.054 (-0.49)	-0.036 (-0.33)	-0.062 (-0.54)	-0.049 (-0.41)	-0.051 (-0.43)
*AR(2)*	0.488	0.507	0.494	0.532	0.486	0.517	0.512
*Sargan*	0.987	0.987	0.991	0.989	0.988	0.993	0.987
*N*	572	572	572	572	572	572	572

The Table presents the results of a dynamic panel data model, the two-step system GMM estimator (Blundell and Bond (1998)), to explain stock market volatility across countries. Standard errors are computed using the finite-sample correction suggested by Windmeijer (2005). The dependent variable is the overall stock market risk of country i in year t. The Voice and Accountability measure (VOICE) captures perceptions of the extent to which a country's citizens are able to participate in selecting their government. The Corruption (CORRUP) measure captures perceptions of the extent to which public power is exercised for private gain. The Government Effectiveness score (GOVEFF) captures perceptions of the quality of public services. Political Stability and Absence of Violence/Terrorism (POLSTAB) captures perceptions of the likelihood that the government will be destabilized or overthrown by unconstitutional or violent means. Regulatory Quality (REGQUAL) captures perceptions of the ability of the government to formulate and implement sound policies and regulations. Rule of Law (RULELAW) captures perceptions of the extent to which agents have confidence in and abide by the rules of society. lnGDPPC denotes the logarithm of the GDP per capita in US dollar. lnTURN is the logarithm of the average daily stock market turnover. RETURN refers to the annualized average daily return of country X within a particular year. FXVOL denotes the annualized standard deviation of daily currency returns vis a vis the dollar. HDI is the United Nations Human Development Index. T-Stats are provided in parentheses. AR(1) and AR(2) refer to the p-values of a test for first- and second-order autocorrelation of the residuals. Sargan refers to the p-values of a test of overidentification.

***, ** and * indicates significance at the 1%, 5% and 10% level, respectively.

Overall, results suggest that a better governance quality (a higher *GI* score) in a particular country can be associated with a less volatile stock market. The better the quality of institutions by which authority in a country is exercised the lower the stock market volatility. Out of the six governance indicators, 3 are statistically significant. In particular, the regulatory quality, the government effectiveness and the control of corruption are important determinants of stock market volatility. At the same time, stock markets in wealthier countries and smaller stock markets, proxied by GDP per capita and turnover, respectively, are less volatile. Furthermore, I observe the well-known negative relationship between returns and volatility. In countries and years of low average returns, stock market volatility is typically higher. In contrast, currency volatility and human development does not seem to explain the differences of stock market volatility across countries. The p values for the Sargan test for overidentification and the test for second order serial correlation in the first-differenced residuals, confirm that there is no problem associated with instrument identification and that the moment conditions cannot be rejected.

Once I decompose overall stock market volatility into a diffusive part and a jump component, my findings suggest that governance primarily affects the jump risk part. Tables 3 and 4 present the results.

**Table 3:** Two-step system GMM estimator, *DIFFURISK*.

*Independent* *Variables*		*VOICE* *(1)*	*RULELAW* *(2)*	*REGQUAL* *(3)*	*POLSTAB* *(4)*	*GOVEFF* *(5)*	*CORRUP* *(6)*
*GOVERNANCE INDICATOR*		-0.004 (-1.16)	-0.004 (-1.27)	-0.006* (-1.69)	-0.001 (-0.93)	-0.006* (-1.71)	-0.005 (-1.60)
*DIFFURISK*_*t-1*_	0.419*** (8.48)	0.432*** (8.82)	0.425*** (9.03)	0.427*** (8.46)	0.418*** (8.11)	0.421*** (8.56)	0.414*** (8.25)
*lnGDPPC*	-0.010* (-1.98)	-0.009 (-1.39)	-0.008 (-1.40)	-0.009* (-1.84)	-0.007 (-1.22)	-0.009 (-1.58)	-0.009* (-1.76)
*lnTURN*	0.001** (2.25)	0.001* (1.85)	0.001 (1.60)	0.001* (1.91)	0.001 (1.59)	0.001* (1.78)	0.001* (1.83)
*RETURN*	-0.006** (-2.28)	-0.008** (-2.40)	-0.007** (-2.18)	-0.008** (-2.32)	-0.006** (-2.09)	-0.007** (-2.29)	-0.006** (-2.15)
*FXVOL*	-0.004 (-0.17)	-0.009 (-0.45)	-0.011 (-0.50)	-0.019 (-0.88)	-0.003 (-0.14)	-0.016 (-0.72)	-0.014 (-0.60)
*HDI*	-0.027 (-0.56)	-0.026 (-0.51)	-0.018 (-0.37)	-0.017 (-0.29)	-0.030 (-0.58)	-0.016 (-0.31)	-0.016 (-0.32)
*AR(2)*	0.405	0.382	0.396	0.366	0.405	0.387	0.373
*Sargan*	0.892	0.896	0.884	0.896	0.893	0.890	0.889
*N*	572	572	572	572	572	572	572

The Table presents the results of a dynamic panel data model, the two-step system GMM estimator (Blundell and Bond (1998)), to explain stock market volatility across countries. Standard errors are computed using the finite-sample correction suggested by Windmeijer (2005). The dependent variable is the diffusive risk part of overall stock market volatility of country i in year t. The Voice and Accountability measure (VOICE) captures perceptions of the extent to which a country's citizens are able to participate in selecting their government. The Corruption (CORRUP) measure captures perceptions of the extent to which public power is exercised for private gain. The Government Effectiveness score (GOVEFF) captures perceptions of the quality of public services. Political Stability and Absence of Violence/Terrorism (POLSTAB) captures perceptions of the likelihood that the government will be destabilized or overthrown by unconstitutional or violent means. Regulatory Quality (REGQUAL) captures perceptions of the ability of the government to formulate and implement sound policies and regulations. Rule of Law (RULELAW) captures perceptions of the extent to which agents have confidence in and abide by the rules of society. lnGDPPC denotes the logarithm of the GDP per capita in US dollar. lnTURN is the logarithm of the average daily stock market turnover. RETURN refers to the annualized average daily return of country X within a particular year. FXVOL denotes the annualized standard deviation of daily currency returns vis a vis the dollar. HDI is the United Nations Human Development Index. T-Stats are provided in parentheses, AR(1) and AR(2) refer to the p-values of a test for first- and second-order autocorrelation of the residuals. Sargan refers to the p-values of a test of overidentification.

***, ** and * indicates significance at the 1%, 5% and 10% level, respectively.

**Table 4:** Two-step system GMM estimator, *JUMPRISK*.

*Independent* *Variables*		*VOICE* *(1)*	*RULELAW* *(2)*	*REGQUAL* *(3)*	*POLSTAB* *(4)*	*GOVEFF* *(5)*	*CORRUP* *(6)*
*GOVERNANCE INDICATOR*		-0.003*** (-2.80)	-0.003** (-2.21)	-0.004*** (-2.97)	-0.001 (-1.56)	-0.004*** (-3.42)	-0.004*** (-3.06)
*JUMPRISK*_*t-1*_	0.187* (1.68)	0.195* (1.79)	0.203* (1.82)	0.202* (1.68)	0.188* (1.71)	0.183* (1.75)	0.185* (1.77)
*lnGDPPC*	0.001 (0.22)	0.001 (0.12)	0.002 (0.34)	0.001 (0.22)	0.001 (0.18)	0.004 (0.70)	0.003 (0.61)
*lnTURN*	-0.001 (-1.24)	-0.001 (-0.64)	-0.001 (-0.80)	-0.001 (-1.05)	-0.001 (-0.95)	-0.001 (-1.09)	-0.001 (-0.98)
*RETURN*	-0.001 (-1.26)	-0.001 (-1.33)	-0.001 (-1.27)	-0.002 (-1.33)	-0.001 (-1.43)	-0.002 (-1.45)	-0.001 (-1.14)
*FXVOL*	0.019* (1.86)	0.015* (1.69)	0.013* (1.81)	0.010* (1.74)	0.020* (1.80)	0.011* (1.79)	0.013* (1.68)
*HDI*	-0.009 (-0.31)	-0.004 (-0.15)	-0.003 (-0.10)	-0.003 (-0.21)	-0.008 (-0.30)	-0.0029 (-0.41)	-0.002 (-0.29)
*AR(2)*	0.221	0.234	0.218	0.235	0.217	0.237	0.239
*Sargan*	0.901	0.905	0.903	0.904	0.905	0.903	0.907
*N*	572	572	572	572	572	572	572

The Table presents the results of a dynamic panel data model, the two-step system GMM estimator (Blundell and Bond (1998)), to explain stock market volatility across countries. Standard errors are computed using the finite-sample correction suggested by Windmeijer (2005). The dependent variable is the jump risk part of overall stock market volatility of country i in year t. The Voice and Accountability measure (VOICE) captures perceptions of the extent to which a country's citizens are able to participate in selecting their government. The Corruption (CORRUP) measure captures perceptions of the extent to which public power is exercised for private gain. The Government Effectiveness score (GOVEFF) captures perceptions of the quality of public services. Political Stability and Absence of Violence/Terrorism (POLSTAB) captures perceptions of the likelihood that the government will be destabilized or overthrown by unconstitutional or violent means. Regulatory Quality (REGQUAL) captures perceptions of the ability of the government to formulate and implement sound policies and regulations. Rule of Law (RULELAW) captures perceptions of the extent to which agents have confidence in and abide by the rules of society. lnGDPPC denotes the logarithm of the GDP per capita in US dollar. lnTURN is the logarithm of the average daily stock market turnover. RETURN refers to the annualized average daily return of country X within a particular year. FXVOL denotes the annualized standard deviation of daily currency returns vis a vis the dollar. HDI is the United Nations Human Development Index. T-Stats are provided in parentheses. AR(1) and AR(2) refer to the p-values of a test for first- and second-order autocorrelation of the residuals. Sargan refers to the p-values of a test of overidentification.

***, ** and * indicates significance at the 1%, 5% and 10% level, respectively.

The regression coefficients presented in Table 3 suggest that governance is typically negative related, but only plays a minor role in explaining diffusive risk. Only the government efficiency and the regulatory quality of a country is significantly explaining the non-jump risk part of overall volatility. Furthermore, diffusive risk is smaller for wealthy countries and smaller stock markets. The well-known negative relationship between returns and volatility is also highly significant for the diffusive part of stock market volatility.

The results presented in Table 4 suggest that governance primarily affect the jump risk part of total stock market volatility, where lower levels of governance quality (a lower score) relate to higher jump risk. The relationship with all governance scores except political stability is highly significant. Among the different governance indicators, the regulatory quality, government efficiency and control of corruption seem to be most important impact. Additionally, only currency volatility is significant and can explain the differences in jump risk across countries. In particular, countries with higher currency volatility experience higher jump risk, which supports the previous findings on the idiosyncratic nature of jump risk.

With respect to my measure for stock market return asymmetries, results suggest that better governance in a particular country can be associated with a more positively skewed distribution of stock market returns, as can be seen in Table 5.

**Table 5:** Two-step system GMM estimator, *SKEWNESS*.

*Independent* *Variables*		*VOICE* *(1)*	*RULELAW* *(2)*	*REGQUAL* *(3)*	*POLSTAB* *(4)*	*GOVEFF* *(5)*	*CORRUP* *(6)*
*GOVERNANCE INDICATOR*		0.009* (1.79)	0.015* (1.89)	0.025** (2.07)	0.010 (1.01)	0.025** (2.01)	0.019* (1.93)
*SKEWNESS*_*t-1*_	0.033* (1.65)	0.035* (1.75)	0.035* (1.67)	0.033* (1.79)	0.032* (1.69)	0.036* (1.66)	0.037* (1.69)
*lnGDPPC*	0.043 (0.97)	0.023 (0.57)	0.017 (0.27)	0.014 (0.31)	0.027 (0.87)	0.020 (0.62)	0.013 (0.19)
*lnTURN*	0.008* (1.76)	0.010* (1.84)	0.001* (1.67)	0.008* (-1.77)	0.008* (-1.66)	-0.008* (-1.73)	-0.008* (-1.70)
*RETURN*	-0.322** (-2.30)	-0.318** (-2.31)	-0.332** (-2.33)	-0.323** (-2.29)	-0.325** (-2.30)	-0.324** (-2.27)	-0.321** (-2.28)
*FXVOL*	-0.456** (-2.46)	-0.420** (-2.32)	-0.419** (-2.21)	-0.403** (-2.15)	-0.457** (-2.24)	-0.375** (-2.02)	-0.384** (-2.06)
*HDI*	-0.252 (-0.98)	-0.196 (-0.80)	-0.235 (-0.87)	-0.297 (-1.17)	-0.263 (-0.98)	-0.217 (-0.90)	-0.225 (-0.90)
*AR(2)*	0.322	0.313	0.319	0.312	0.324	0.314	0.314
*Sargan*	0.889	0.898	0.894	0.893	0.899	0.897	0.885
*N*	572	572	572	572	572	572	572

The Table presents the results of a dynamic panel data model, the two-step system GMM estimator (Blundell and Bond (1998)), to explain stock market skewness across countries. Standard errors are computed using the finite-sample correction suggested by Windmeijer (2005). The dependent variable is my measure of return asymmetry in country i’s stock market in year t. The Voice and Accountability measure (VOICE) captures perceptions of the extent to which a country's citizens are able to participate in selecting their government. The Corruption (CORRUP) measure captures perceptions of the extent to which public power is exercised for private gain. The Government Effectiveness score (GOVEFF) captures perceptions of the quality of public services. Political Stability and Absence of Violence/Terrorism (POLSTAB) captures perceptions of the likelihood that the government will be destabilized or overthrown by unconstitutional or violent means. Regulatory Quality (REGQUAL) captures perceptions of the ability of the government to formulate and implement sound policies and regulations. Rule of Law (RULELAW) captures perceptions of the extent to which agents have confidence in and abide by the rules of society. lnGDPPC denotes the logarithm of the GDP per capita in US dollar. lnTURN is the logarithm of the average daily stock market turnover. RETURN refers to the annualized average daily return of country X within a particular year. FXVOL denotes the annualized standard deviation of daily currency returns vis a vis the dollar. HDI is the United Nations Human Development Index. T-Stats are provided in parentheses. AR(1) and AR(2) refer to the p-values of a test for first- and second-order autocorrelation of the residuals. Sargan refers to the p-values of a test of overidentification.

***, ** and * indicates significance at the 1%, 5% and 10% level, respectively.

Again, poorly governed countries experience more negative jumps and/or a higher intensity of jumps, leading to more negatively skewed return distributions. The relationship with all governance scores except political stability is significant. Again, among the different governance indicators, the regulatory quality, government efficiency and control of corruption have the strongest impact. Interestingly, the relationship with GDP per capita and the *HDI* measure is insignificant, further suggesting that income differences and differences in human development across countries do not explain jumps in stock markets. Market turnover is only marginally significant. However, a higher average market return and higher currency volatility in a particular country can be associated with more negative jumps and, therefore, more negative return asymmetry. Again, The p values for the Sargan test for overidentification and the test for second order serial correlation in the first-differenced residuals, confirm that there is no problem associated with instrument identification and that the moment conditions cannot be rejected.

## Conclusions

Governance or the management of government institutions varies substantially across countries. The literature suggests that good governance has a positive effect on the development of financial markets and equity returns, in particular; it lowers equity volatility and, therefore, the costs of equity financing. However, previous literature provides little guidance about asset pricing implications of governance. Additionally, the international nature of price jumps in stock markets and its relationship with country characteristics is not well understood. Price jumps are prevalent in stock markets all over the world, but jumps are found to be far less systematic than the smooth (non-jump) component of country price indexes. Hence, if jumps are more idiosyncratic, governance should primarily affect the jump risk component of stock market volatility. This is good news for international investors, because diversification provides insurance against jumps. Relying on an equilibrium asset-pricing model in an economy under jump diffusion, I decompose the moments of the returns of international stock markets into a diffusive (systematic) risk and a (idiosyncratic) jump risk part. Using stock market data for a balanced panel of 52 countries, my results suggest that the regulatory quality, the government effectiveness and the control of corruption are important determinant of (idiosyncratic) jump risk. Stock markets in poorly governed countries are characterized by higher volatility and more negative return asymmetry, primarily driven by the higher jump risk. Results are robust to the inclusion of various controls for other country- or market-specific characteristics. Therefore, my results lend further support for the view that a precondition for financial market development is the improvement of the government institutions.
